# Virtual Patients for Communication Skills Training: A Mixed Methods Evaluation

**DOI:** 10.7759/cureus.91005

**Published:** 2025-08-26

**Authors:** Maidha Jadoon, Khurram Naushad, Fatima Aman, Yasir Khan, Mushyyada Durrani, Shagufta Ali

**Affiliations:** 1 Department of Medical Education and Research, Women Medical College, Abbottabad, PAK; 2 Department of Medical Education, Khyber Girls Medical College, Peshawar, PAK; 3 Department of Medical Education, Saidu Medical College, Swat, PAK; 4 Department of Dental Education, Peshawar Dental College, Peshawar, PAK; 5 Department of Medical Education, Jinnah Medical College, Peshawar, PAK

**Keywords:** communication training, computer simulation, learner experience, medical education, mixed methods, physician-patient relations, simulation-based learning, virtual patients

## Abstract

Background: Effective communication is critical in clinical practice, and virtual patients offer a promising, safe alternative for enhancing communication training in medical education.

Objective: To evaluate the effectiveness of virtual patient-based simulation in enhancing key communication skills-such as active listening, empathy, and clarity of explanations-and to explore learner experience, including engagement, satisfaction, usability, and perceived educational value, using a mixed methods approach.

Materials and Methods: This one-year mixed-methods study (Aug 2022-Jul 2023) assessed the effectiveness and learner experience of virtual patient-based communication training among undergraduate and postgraduate medical trainees across six institutions in Pakistan. A total of 258 undergraduate and postgraduate medical trainees participated in virtual simulations using the Body Interact™ platform (Take The Wind, SA, Coimbra, Portugal). Participants were enrolled through convenience sampling and represented a mix of clinical-year undergraduates and early- to mid-stage postgraduate trainees, with no prior formal training in virtual patient-based communication tools. Quantitative data were collected through pre- and post-training questionnaires assessing communication skills, confidence, and satisfaction, analyzed using paired t-tests. Qualitative data were obtained via semi-structured interviews with a subset of participants (n ≈ 25) and analyzed using thematic analysis. Triangulation integrated both data sets for comprehensive interpretation.

Results: Participants showed significant improvement in communication domains, including active listening (pre: 3.21±0.81; post: 4.11±0.66; *p*<0.001), empathy and rapport (pre: 3.05±0.76; post: 4.00±0.61; *p*<0.001), and clarity of explanations (pre: 3.14±0.83; post: 4.02±0.59; *p*<0.001). Overall communication confidence increased by +1.16 points (*p*<0.001), and satisfaction by +1.15 points (*p*<0.001). Qualitative themes supported these findings, highlighting perceived realism, engagement, and reflective learning, with minor usability concerns.

Conclusion: Virtual patients are an effective and engaging tool for improving healthcare communication skills in clinical trainees.

## Introduction

Effective communication is fundamental to high-quality healthcare delivery [1]. From accurately eliciting patient histories to conveying diagnoses and discussing treatment options, healthcare professionals rely heavily on their communication skills to ensure patient satisfaction, adherence to treatment, and overall clinical outcomes [2,3]. Despite its importance, traditional communication training in medical education often faces challenges such as limited patient access, time constraints, variability in teaching quality, and difficulty in providing consistent, objective feedback [4]. In this context, innovative educational tools are being explored to enhance the development of communication skills among healthcare trainees [5].

One such innovation is the use of virtual patients, computer-generated simulations that replicate real-life clinical encounters [6]. These digital platforms offer standardized, repeatable scenarios in a safe, controlled environment, allowing learners to practice communication strategies without the risk of harming actual patients [7]. Virtual patients can be programmed to display a wide range of emotional and clinical presentations, facilitating exposure to diverse clinical situations that may be difficult to encounter in routine training [8]. Furthermore, some systems provide real-time feedback or post-interaction analysis, enhancing reflective learning and self-assessment [9].

Emerging research suggests that virtual patient technologies may positively impact learning outcomes, engagement, and skill acquisition [10]. However, the effectiveness of virtual patients in training communication, especially in comparison with traditional methods or in blended learning formats, remains an evolving area of inquiry [11]. Factors such as authenticity, user experience, and integration into curricula are critical for maximizing their educational value. Additionally, understanding how learners perceive and interact with virtual patients can inform further development of these tools and their alignment with pedagogical goals [12].

To contribute to this evolving field, the present study employs a mixed methods design to explore the utility of virtual patients in healthcare communication training. By integrating both quantitative and qualitative approaches, the research seeks to provide a comprehensive understanding of the effectiveness, usability, and experiential impact of virtual patient-based learning environments. Specifically, the study aimed to evaluate the impact of virtual patient-based simulation on key communication skills, such as active listening, empathy, and clarity of explanations, among medical trainees, and to explore their learner experience, including engagement, satisfaction, perceived realism, and educational value, using a mixed-methods approach.

## Materials and methods

Study design and setting

This study employed a mixed-methods design to evaluate the effectiveness and learner experience of virtual patients in healthcare communication training among undergraduate and postgraduate medical trainees. The research was conducted across multiple institutions in Pakistan, including the Department of Medical Education and Research at Women Medical College (Abbottabad), the Department of Medical Education at Khyber Girls Medical College (Peshawar), the Department of Medical Education at Saidu Medical College (Swat), the Department of Dental Education at Peshawar Dental College (Peshawar), the Department of Medical Education at Jinnah Medical College (Peshawar), and the postgraduate and undergraduate training programs at Hayatabad Medical Complex (Peshawar). The study was carried out over a period of one year, from August 2022 to July 2023. It combined quantitative surveys with qualitative feedback to gain comprehensive insights into the educational impact of virtual patient simulations. The virtual simulations were conducted using the Body Interact™ platform (Take The Wind, SA, Coimbra, Portugal), a widely used virtual patient software that provides interactive, case-based clinical scenarios tailored for communication training. The simulation scenarios used in training focused on common clinical situations involving doctor-patient communication, including history taking, breaking bad news, and shared decision-making. Each case lasted approximately 15-20 minutes and was selected by medical educators based on relevance to communication skills development. The inclusion and exclusion criteria are summarized in Table 1.

**Table 1:** Inclusion and Exclusion Criteria

Criteria Type	Criterion
Inclusion	Undergraduate and postgraduate medical trainees enrolled in clinical rotations
	Trainees with access to the virtual patient communication module during the study period
	Provided informed consent
	Completed the full training module
Exclusion	Prior formal training in virtual patient-based communication tools
	Failed to complete the simulation exercises
	Unable to access the platform due to technical issues

Sample size

A total of 258 participants were enrolled using convenience sampling from eligible undergraduate and postgraduate medical trainees across six institutions in Pakistan. The sampling approach was chosen due to the multi-institutional academic setting and the intent to include all consecutive trainees who met the inclusion criteria and provided informed consent during the one-year study period (August 2022-July 2023). No formal a priori power or sample size calculation was conducted, as the study was exploratory in nature and aimed to reflect real-world educational practices in communication training. Inclusion continued until the end of the study period without a pre-defined numerical cutoff. Of the total participants, 172 (66.7%) were clinical-year undergraduate students and 86 (33.3%) were postgraduate trainees. None had prior formal training in virtual patient-based communication tools, in accordance with the study's exclusion criteria. Although the lack of a formal sample size calculation is a limitation, the final sample is comparable to similar educational studies, as referenced in Borja-Hart et al. [13] and Sezer et al. [14].

Data collection

Quantitative data were collected through structured pre- and post-training questionnaires assessing communication skills, learner confidence, and satisfaction, using a 5-point Likert scale [15]. The scale was anchored as follows: Strongly Disagree = 1; Disagree = 2; Neither Agree nor Disagree = 3; Agree = 4; Strongly Agree = 5. The questionnaire was developed specifically for this study in line with established educational research practices. It was pilot-tested on a group of trainees (n = 10) to ensure clarity, relevance, and face validity. Internal consistency was satisfactory (Cronbach’s alpha > 0.8 across domains), supporting its reliability for use in this context. To gain deeper insights into learner experiences, qualitative data were gathered through semi-structured interviews and open-ended feedback forms. A purposive sampling strategy was employed to select a diverse subset of participants (n ≈ 25) for the qualitative component, ensuring representation across undergraduate and postgraduate training levels, gender, and participating institutions. Participants were invited following completion of the training based on their availability and willingness to provide detailed feedback. The qualitative component aimed to explore in-depth perceptions, usability, and engagement with virtual patient simulations. Key prompts used in the semi-structured interviews are included in the supplementary material. Interviews were conducted by trained qualitative researchers unaffiliated with the participating institutions and were audio-recorded with participant consent. Thematic saturation was reached after 22 interviews; three additional interviews were conducted to confirm saturation.

Thematic analysis was conducted using Braun and Clarke’s six-phase framework [16], which involved familiarization with the data, generation of initial codes, searching for themes, reviewing themes, defining and naming themes, and producing the final report. Triangulation of data followed the side-by-side comparison approach described by Fetters et al. [17], aligning qualitative themes with quantitative trends for integrated interpretation. Triangulation was achieved by integrating quantitative findings (e.g., improvements in confidence and satisfaction scores) with qualitative themes (e.g., perceived realism, usability barriers), enabling cross-validation and a more comprehensive interpretation of the results.

Statistical analysis

Quantitative data were analyzed using SPSS version 26 (IBM Corp., Armonk, NY). Descriptive statistics were computed for demographic variables and survey responses. Paired t-tests were applied to compare pre- and post-intervention scores. A p-value < 0.05 was considered statistically significant. Thematic analysis results were compared and cross-referenced with quantitative trends to strengthen the interpretation through triangulation.

Ethical considerations

This study was approved by the Hospital Research and Ethical Committee, Medical Teaching Institute (MTI) - Hayatabad Medical Complex, Peshawar (Approval No. 452, dated 10 August 2022). Permission to conduct the study was also obtained from the heads of departments of all participating institutions. Written informed consent was obtained from all participants prior to data collection, and confidentiality and anonymity of the participants were maintained throughout the study.

## Results

Out of the 258 participants, 132 (51.16%) were male and 126 (48.84%) were female (Table 2). The majority were undergraduate MBBS trainees (n = 176, 68.22%), while 82 participants (31.78%) were enrolled in postgraduate training programs (FCPS/MD). Most participants (n = 217, 84.11%) reported no prior simulation experience, whereas only 41 (15.89%) had previously engaged in general clinical or procedural simulations, such as skills labs or mannequin-based training. Importantly, none of the participants had formal training or experience with virtual patient platforms specifically designed for communication training. This distinction confirms adherence to the study’s exclusion criteria, which excluded individuals with prior exposure to communication-focused simulation tools. Regarding departmental distribution, participants were primarily affiliated with Internal Medicine (n = 74; 28.68%), followed by Surgery (n = 63; 24.42%), Pediatrics (n = 48; 18.60%), Gynecology and Obstetrics (n = 40; 15.50%), and other departments (n = 33; 12.79%).

**Table 2:** Demographic Characteristics of Participants

Variable	Category	Number of Participants (n)	Percentage (%)
Gender	Male	132	51.16
	Female	126	48.84
Level of Training	Undergraduate (MBBS)	176	68.22
	Postgraduate (FCPS/MD)	82	31.78
Prior Simulation Experience	Yes	41	15.89
	No	217	84.11
Department/Rotation	Internal Medicine	74	28.68
	Surgery	63	24.42
	Pediatrics	48	18.60
	Gynecology & Obstetrics	40	15.50
	Other	33	12.79

There were statistically significant improvements across all measured communication domains after the virtual patient training (p < 0.001), shown in Table 3. Active listening increased from a mean of 3.21 to 4.11 (+0.90), empathy and rapport from 3.05 to 4.00 (+0.95), clarity of explanations from 3.14 to 4.02 (+0.88), and patient engagement from 2.98 to 3.89 (+0.91). The overall communication score rose from 3.10 to 4.00, showing a substantial mean difference of +0.90.

**Table 3:** Pre- and Post-training Communication Skills Scores Scores measured on a 5-point Likert scale (1 = strongly disagree, 2 = disagree, 3 = neither agree nor disagree, 4 = agree, 5 = strongly agree). Values are presented as mean (SD). Differences analyzed using paired t-test; p < 0.05 considered statistically significant.

Communication Domain	Pre-training Mean (SD)	Post-training Mean (SD)	Mean Difference	p-value
Active Listening	3.21 (0.81)	4.11 (0.66)	+0.90	< 0.001
Empathy & Rapport	3.05 (0.76)	4.00 (0.61)	+0.95	< 0.001
Clarity of Explanations	3.14 (0.83)	4.02 (0.59)	+0.88	< 0.001
Patient Engagement	2.98 (0.91)	3.89 (0.73)	+0.91	< 0.001
Overall Communication Score	3.10 (0.78)	4.00 (0.61)	+0.90	< 0.001

Post-training results showed significant increases in learner confidence and satisfaction (Table 4). Overall communication confidence improved from 3.10 ± 0.72 to 4.26 ± 0.57 (+1.16), and satisfaction with training rose from 3.15 ± 0.70 to 4.30 ± 0.61 (+1.15), both with p-values < 0.001. Additionally, participants expressed strong endorsement of the training, with a mean recommendation score of 4.32 ± 0.55.

**Table 4:** Learner Confidence, Satisfaction, and Recommendation Scores measured on a 5-point Likert scale (1 = strongly disagree, 2 = disagree, 3 = neither agree nor disagree, 4 = agree, 5 = strongly agree). Values are presented as mean ± SD. Higher scores indicate greater confidence, higher satisfaction, and stronger likelihood of recommending the training to others. Differences between pre- and post-training scores were analyzed using paired t-test; p < 0.05 considered statistically significant.

Measure	Pre-training Mean ± SD	Post-training Mean ± SD	Mean Difference	p-value
Overall Communication Confidence	3.10 ± 0.72	4.26 ± 0.57	+1.16	<0.001
Satisfaction with Training	3.15 ± 0.70	4.30 ± 0.61	+1.15	<0.001
Recommendation to Others	—	4.32 ± 0.55	—	—

Thematic analysis of qualitative data (n ≈ 25) revealed five key themes: perceived realism, with learners describing virtual patients as emotionally and clinically lifelike; a safe learning environment enabling practice without real-patient risk; usability challenges such as interface issues; enhanced reflective learning through system feedback; and increased engagement due to the interactive and immersive nature of the modules (Table 5).

**Table 5:** Themes from Qualitative Analysis (n=25) Values represent the number of participants (n) who contributed to each theme. Some participants expressed more than one theme, so totals exceed n = 25.

Theme No.	Theme Title	Description	Representative Quote	Participants n (%)
1	Perceived Realism	Virtual patients felt lifelike and presented realistic emotional and clinical responses.	“The virtual patient’s expressions and language felt real.”	18 (72%)
2	Safe Learning Environment	Learners appreciated being able to practice without fear of harming real patients.	“I could make mistakes and learn without stress.”	16 (64%)
3	Usability Challenges	Technical or navigation issues occasionally disrupted the experience.	“Sometimes the interface was slow or confusing.”	10 (40%)
4	Enhanced Reflective Learning	Feedback and performance tracking promoted reflection on communication style.	“The feedback made me think about how I explain things.”	14 (56%)
5	Increased Engagement	Interactive nature kept learners attentive and interested throughout the module.	“It was much more engaging than traditional lectures.”	20 (80%)

The triangulated results demonstrated alignment between quantitative improvements and qualitative insights. Increased communication scores matched themes of perceived realism; higher confidence aligned with the safe learning environment; satisfaction correlated with increased engagement; and improved clarity of explanations reflected enhanced reflective learning (Table 6). Usability challenges appeared in qualitative data but had minimal quantitative impact, indicating they were not widespread.

**Table 6:** Triangulation of Quantitative and Qualitative Findings

Quantitative Domain	Qualitative Theme	Integrated Interpretation	Representative Quote
Overall Communication Scores	Perceived Realism	Improvement in measured communication skills aligns with learners’ sense of lifelike interaction.	“The virtual patient’s expressions and language felt real.”
Confidence in Communication	Safe Learning Environment	Increased confidence correlates with learners feeling safe to experiment without risk.	“I could make mistakes and learn without stress.”
Satisfaction with Training	Increased Engagement	High satisfaction levels match reports of high learner interest and attentiveness.	“It was much more engaging than traditional lectures.”
Clarity of Explanation Scores	Enhanced Reflective Learning	Improvement in clarity reflects learners' ability to self-evaluate through system feedback.	“The feedback made me think about how I explain things.”
Slight Usability Concerns Noted	Usability Challenges	A minority of participants reported technical barriers, reflected in qualitative responses but not major in statistics.	“Sometimes the interface was slow or confusing.”

## Discussion

The present mixed-methods study evaluated the effectiveness and learner experience of virtual patients in healthcare communication training. Our findings revealed statistically significant improvements in multiple communication domains. For instance, active listening improved from a pre-training mean score of 3.21 to 4.11 post-training (+0.90, p < 0.001), while empathy and rapport rose from 3.05 to 4.00 (+0.95, p < 0.001). These outcomes are consistent with previous studies that demonstrated enhanced communication competency following virtual patient-based training. Cook et al. [18] reported similar improvements in empathy and information delivery among medical students using virtual patients in simulated interviews. These improvements highlight the value of structured simulation exercises in addressing common communication gaps among trainees. Such findings reinforce the growing advocacy for incorporating virtual simulation into standard medical curricula to complement traditional teaching methods.

Learner confidence and satisfaction also increased substantially, with overall communication confidence rising from 3.10 ± 0.72 to 4.26 ± 0.57 (+1.16, p < 0.001), and satisfaction with training increasing from 3.15 ± 0.70 to 4.30 ± 0.61 (+1.15, p < 0.001). These findings align with a study by Pence et al. [19], where 78% of learners reported enhanced self-confidence and satisfaction following virtual patient sessions. Our high post-training recommendation score (4.32 ± 0.55) also supports previous evidence that virtual patient technology is perceived as a valuable and acceptable pedagogical tool [20]. Increased confidence among learners suggests that virtual patient-based training helps reduce anxiety in real clinical encounters. Furthermore, high satisfaction scores indicate a positive learner perception, which is crucial for the adoption and sustainability of such innovative educational interventions.

Qualitative data further enriched our understanding by highlighting themes such as perceived realism and a safe learning environment. Learners reported that "the virtual patient’s expressions and language felt real," reinforcing the authenticity of the virtual patient experience. This perception aligns with the previous findings, which emphasized that emotional engagement and realistic scenarios in simulation-based learning environments enhance learner involvement and may support the transfer of communication and interpersonal skills to clinical practice [21]. Such realism is particularly important in developing empathy and rapport, key components of effective communication. The psychologically safe environment also encourages active experimentation and fosters deeper learning without fear of judgment or harm.

The integration of qualitative and quantitative findings revealed strong triangulation. For example, the improvement in clarity of explanations (from 3.14 to 4.02, +0.88, p < 0.001) was complemented by the theme of enhanced reflective learning. Learners indicated that feedback mechanisms encouraged self-assessment, consistent with the previous research work, which demonstrated that reflection in simulation settings reinforces long-term communication improvements [22]. This alignment between numerical gains and thematic insights strengthens the validity of the findings. It also illustrates how mixed methods designs provide a more holistic understanding of educational interventions compared to single-method approaches.

Although some usability challenges were noted (e.g., interface issues), these were infrequent and did not significantly affect the overall outcome. Technical limitations were primarily related to navigation difficulties and occasional lag in simulation response. Despite these minor barriers, learners expressed a willingness to continue using the platform and recommended its wider implementation. Addressing such usability concerns through improved training and platform updates could further enhance the learner experience. It is also crucial to consider accessibility and user-friendliness in future iterations of virtual simulation tools to maximize their educational potential.

These findings emphasize the educational value of virtual patients in communication training, echoing similar evidence from various international settings. The demonstrated improvements in communication domains, confidence, and learner satisfaction collectively affirm the value of virtual patient-based simulations as a complement to traditional teaching methods. Future studies could explore long-term retention of skills acquired through virtual patient training and its impact on actual clinical performance. Continued research in this area may also help identify the most effective implementation strategies tailored to different learner populations and cultural contexts.

Strengths and limitations

A major strength of this study lies in its mixed methods design, which allowed for a comprehensive evaluation of both the measurable outcomes and experiential aspects of virtual patient training. The integration of quantitative improvements - such as a +0.90 increase in overall communication scores and a +1.16 rise in learner confidence - with rich qualitative feedback provided a well-rounded understanding of virtual patient effectiveness. The use of a validated platform (Body Interact™), a relatively large sample size (n = 258), and real-world clinical trainees from diverse rotations further enhance the study's applicability. The study also ensured diversity in participant demographics across training levels, gender, and departments, and employed purposive sampling for qualitative interviews to ensure broad representation.

However, the study is limited by its single-center setting and use of convenience sampling, which may affect generalizability. The absence of a control group and lack of long-term follow-up restrict our ability to assess sustained impact and compare outcomes with traditional training methods. Additionally, no formal sample size calculation was conducted due to the exploratory nature of the study. Technical issues reported by a minority of participants also indicate a need for further optimization of the user interface and accessibility. While triangulation was used to integrate qualitative and quantitative data, future studies may benefit from applying a formal mixed methods integration framework from the outset. Greater detail on virtual patient scenario design and expanded usability testing may also enhance tool development and learner experience.

## Conclusions

This study demonstrates that virtual patient-based training is associated with significant self-reported improvements in communication skills, learner confidence, and satisfaction among medical trainees. The substantial pre- to post-training gains in active listening, empathy, rapport, and clarity of explanations highlight the perceived educational value of this modality. Qualitative feedback further supported these findings, with participants emphasizing the realism, engagement, and reflective learning fostered by virtual patient simulations. However, these improvements represent perceived gains rather than objectively measured performance changes. These findings support the integration of virtual patients into medical curricula as a complementary tool to traditional communication training, rather than a standalone replacement. Future studies should incorporate objective assessments, control groups, and longitudinal follow-up to evaluate the sustained impact and actual performance outcomes of virtual patient-based communication training.
